# Minimally invasive technique for the abscess drainage in lumbosacral tuberculosis using arthroscopy sheath: A case report

**DOI:** 10.1016/j.ijscr.2020.04.007

**Published:** 2020-05-19

**Authors:** Singkat Dohar Apul Lumban Tobing, Dody Kurniawan

**Affiliations:** a Department of Orthopaedic and Traumatology, Faculty of Medicine Universitas Indonesia, Dr. Cipto Mangunkusumo Hospital, Jakarta, Indonesia; b Resident of Department of Orthopaedic and Traumatology, Faculty of Medicine Universitas Indonesia, Dr. Cipto Mangunkusumo Hospital, Jakarta, Indonesia

**Keywords:** Lumbosacral tuberculosis, Minimally invasive surgery, Arthroscopy, Debridement, Abscess evacuation

## Abstract

**Introduction:**

Spinal tuberculosis usually affects the thoracolumbar spine, with only 2–3% involving the lumbosacral region. Lumbosacral tuberculosis can lead to the formation of a presacral abscess. For drainage of the spinal abscess, the presacral region is one of the problematic regions to perform. Minimally invasive surgery (MIS) is an essential clinical technique for the debridement of the spinal abscess in order to decrease the morbidity acquired by the patient. We presented a case of lumbosacral tuberculosis treated with abscess evacuation using the MIS technique.

**Presentation of case:**

A 28-year-old male came with the chief complaint of back pain and a lump in the right groin area for four months before admission. Physical examination showed a lump and bilateral positive straight leg raising. Preoperative Oswestry Disability Index (ODI) and the Japanese Orthopaedic Association (JOA) scores showed moderate disability and normal function, respectively. Radiologic examinations showed signs of lumbosacral tuberculosis. The patient underwent abscess evacuation using MIS of presacral approach as described for axial lumbar interbody fusion (AxiaLIF), and improvement in ODI score was noted.

**Discussion:**

A presacral approach, as what we performed, is a simple approach that can reach the location of the abscess by using fluoroscopic guidance. Abscess evacuation was confirmed by the presence of clear fluid, which indicated that the caseous material had been removed thoroughly, and also, as the clear fluid was no longer noticed.

**Conclusion:**

The success of this approach depends on the time of surgery. When surgery is delayed, the granulation tissue has been formed, making minimally invasive techniques for surgical evacuation much more difficult.

## Highlights

• Minimally invasive surgery using a presacral approach and AxiaLIF is safe and can be an option for evacuation of paraspinal abscesses.
• Early diagnosis and early treatment avoid alteration of the liquid abscess into granulomatous tissue.
• Preoperative prerequisites must be fulfilled.
• Minimally invasive surgery is favored as extensive soft tissue is avoided and with lesser blood loss.

## 1 Introduction

The estimated prevalence of TB infection in Indonesia reached more than 300 new cases per year [1]. This number is predicted to stay high especially in population who have co-infection with HIV and diabetes [2]. Tuberculosis (TB) of the spine, also known as Pott’s disease, is one of the most common extrapulmonary TB infection after tuberculosis of the lymph nodes, comprising 50% of all skeletal TB [3]. The lesion usually affects the thoracolumbar spine, with only 2–3% involving the lumbosacral region [4]. Infection in this region carries specific characteristics including less risk of kyphosis due to the lordotic nature of the lumbosacral spine [5]. However, the patients usually end up with hypolordosis or straightening of the spine. This condition has been showed to cause postoperative back pain and complication during the upcoming pregnancy [6].

The management of spinal TB has been primarily involved chemotherapy and surgical drainage [7]. Conservative treatment is appropriate in most cases where there is no evidence of progressive bony destruction, improper healing, persistent pain, deteriorating neurological condition, or profound deformity of instability [8]. Lumbosacral tuberculosis can lead to the formation of a presacral abscess. The indication of the drainage of such abscess is presence of pressure symptom or if the abscess does not regress with anti-tuberculous treatment. For drainage of spinal abscess, presacral region is one of the problematic regions to perform [9].

If lumbosacral tuberculosis has signs of abscesses, cavities, sequestra, and sinus formation, routine treatment by thorough debridement is recommended, along with bone graft and/or internal fixation. The development of surgical techniques has brought a minimally invasive surgery (MIS) as an essential clinical technique. In the debridement of this lumbosacral abscess, we used MIS to reduce the morbidity acquired by the patient [10]. This case report had been written according to the SCARE guideline [11].

## 2 Patient information

A 28-year-old male was seen by the digestive surgery for a lump on the right groin area with a back pain (VAS 5) for the past four months. Because there was no abnormality in abdomen area, the patient was consulted to Orthopaedic Department. The pain did not radiate and was relieved when the patient lies down on his back. There was no numbness or tingling sensation. He had no trouble with micturition and defecation. The patient did not have a history of lung tuberculosis. However, the patient was sent to the pulmonary division before our department and was diagnosed with tuberculous spondylitis and was given anti-tuberculous drugs since then. The clinical outcomes were assessed preoperatively and at the final follow-up by Oswestry Disability Index (ODI) and Japanese Orthopaedic Association (JOA) scores. The preoperative ODI score for this patient was categorized as moderate disability, whereas the JOA score for this patient is in normal function state.

## 3 Clinical findings

**Fig. 1:**
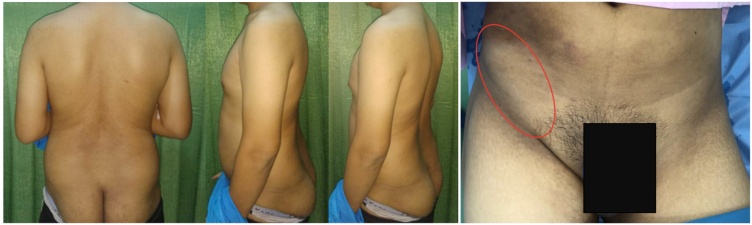
Physical Examination of the Back.

**Fig. 2:**
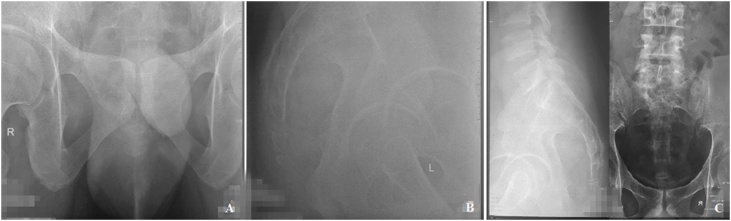
Pelvis and Lumbosacral Plain Radiograph of the Patient.

Examination of the back showed no apparent deformities (Fig. 1); however, the range of motion of the back was decreased due to pain. On the right groin area, a 7 × 4 × 1 cm mass with cystic consistency was visible (Fig. 2). It was painful on palpation. The examination of the abdomen showed no other pathologies. Single leg raise test of both legs elicited pain at 20 degrees ROM. The neurological examination was otherwise normal.

## 4 Diagnostic assessment

**Fig. 3:**
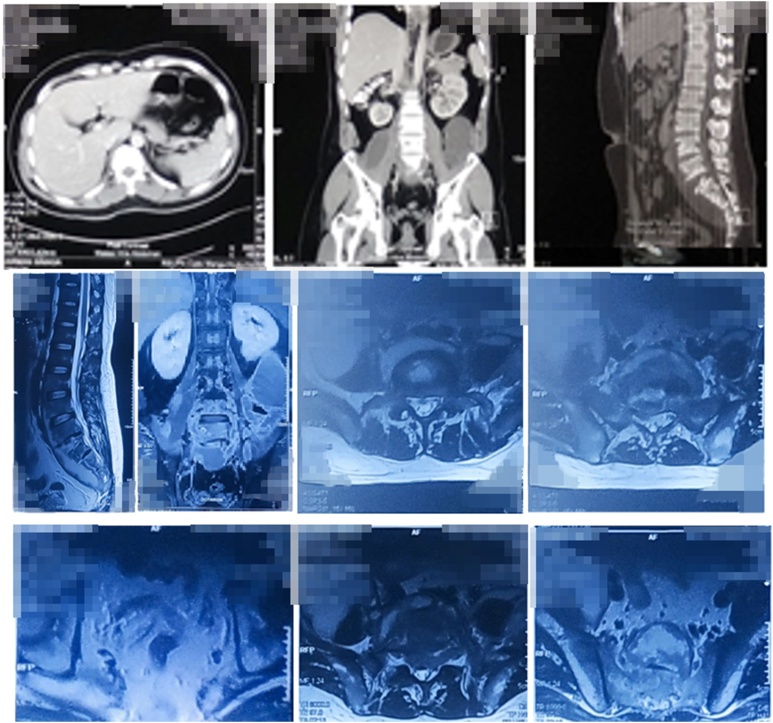
Lumbosacral CT and MRI of the Patient.

**Fig. 4:**
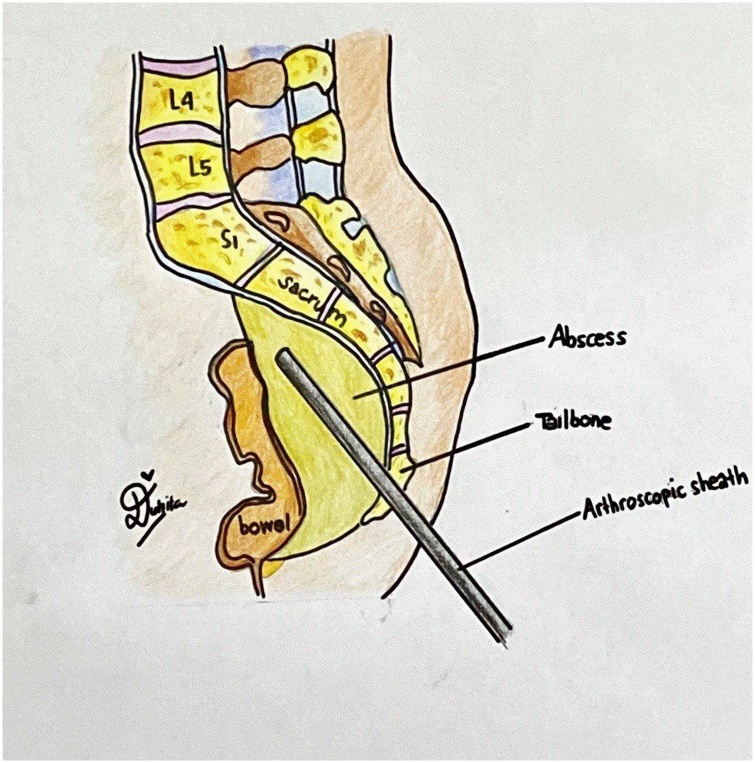
The Illustration of the AxiaLIF Approach as Described.

**Fig. 5:**
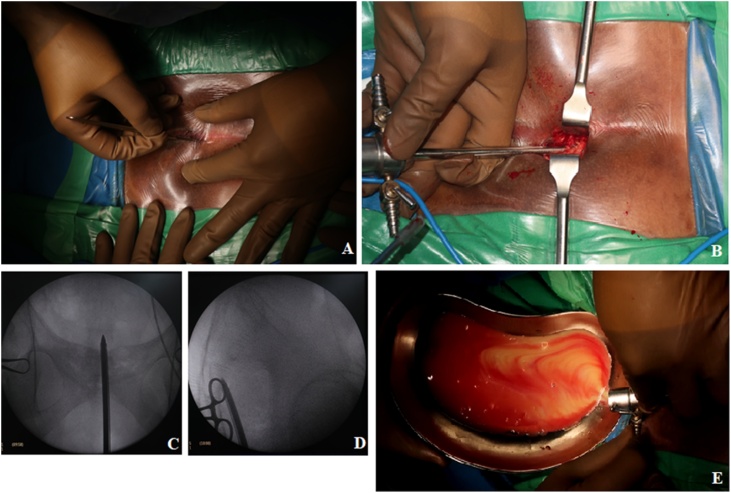
Intraoperative Procedures.

A standard AP and lateral plain radiograph of the pelvis and lumbosacral revealed erosion of the anterior aspect of L4–L5, which was accompanied by psoas haziness (Fig. 3). CT scan of the abdomen showed spondylodiscitis of L3-S5 with paravertebral abscess formation extending to the right psoas muscle and inguinal region (Fig. 4). Evidence of paravertebral abscess was further confirmed by contrast-enhanced MRI (Fig. 5). Blood works showed elevation of Complete Blood Count and ESR. Molecular GeneXpert test proved the infection of *Mycobacterium tuberculosis*.

## 5 Therapeutic intervention

The patient was diagnosed with spondylitis TB of L3-S5 with paravertebral abscess. The patient was put on 1st line TB medication (RHZE) and underwent anterior debridement and abscess evacuation. The tuberculous medications consisted of 4-FDC (Rifampin 150 mg, Isoniazid 75 mg, Pyrazinamide 400 mg, and Ethambutol HCL 275 mg, which was started three weeks before surgery and continued after surgery.

**Fig. 6:**
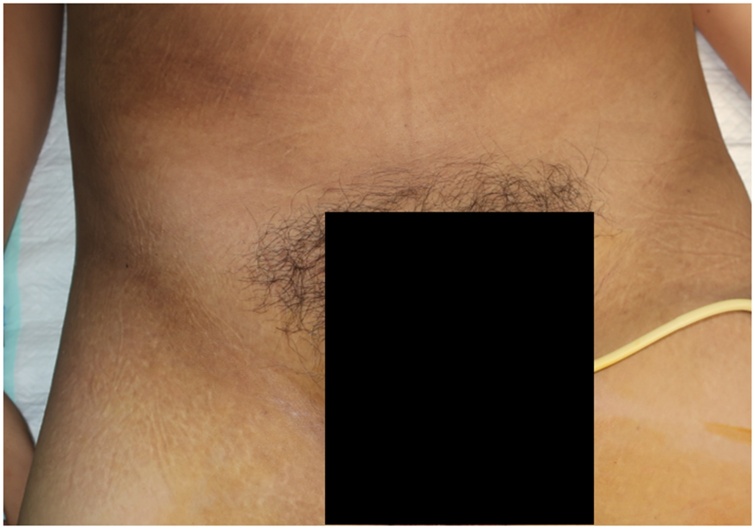
Postoperative Patient Physical Examination.

Surgery performed to the patient was anterior debridement and abscess evacuation through minimally invasive surgery (MIS) of using arthroscopy sheath with a blunt trocar. The approach used in this MIS is the approach used to apply AxiaLIF system, as described by Rapp et al. [12] (Fig. 6). The patient received endotracheal intubation under general anesthesia in a prone position.

**Fig. 7:**
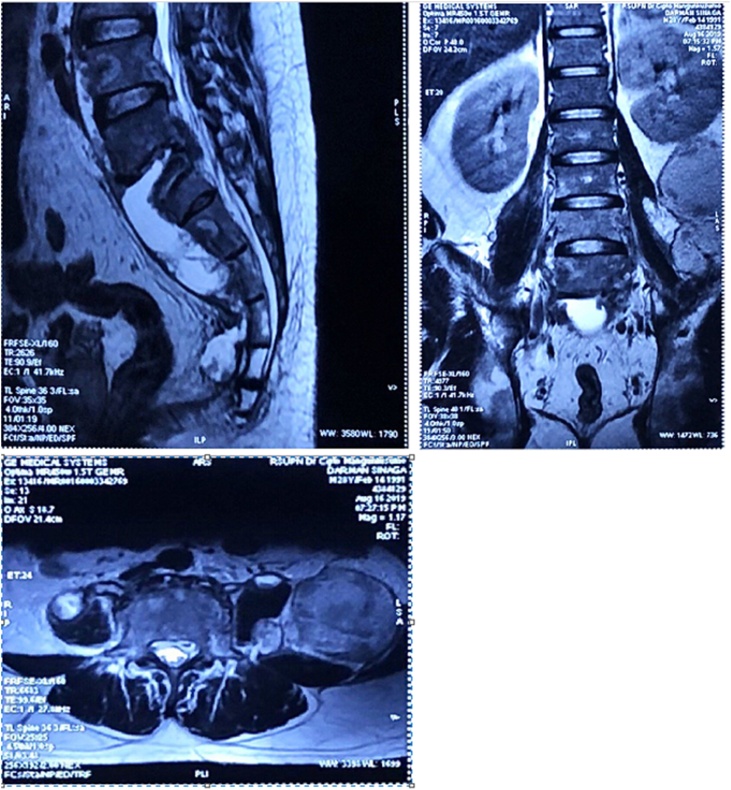
Patient MRI Following the Procedure.

This procedure was performed entirely under the guidance of the fluoroscopy without direct visualization of the operative field. The surgery was started by creating a 2 cm longitudinal incision at the level of the paracoccygeal notch or tip of the coccyx (presacral approach). Subsequently, the arthroscopy sheath with blunt trocar was advanced through the presacral space and stay aimed at the midline, which is avascular, and was pushed into the sacrum in the location of the abscess. The entry point of this arthroscopy sheath is lateral to the coccyx and inferior to the attachment of the sacrospinous and sacrotuberous ligament. Then, a large-sized blunt arthroscopic trochar of 4.0 × 175 mm was inserted along the same pathway, climbing up to the anterior sacral cortex and then progressed 0.5 cm anterior to anterior sacral body and into the abscess (Fig. 7). After the trochar achieves the abscess, the abscess was drained. Around 1 L of pus mixed with caseous materials was evacuated from the abscess. Warm normal saline was used to irrigate and drain the abscess thoroughly until the withdrawn fluid became clear. This clear fluid coming out from the drainage system confirmed the completeness of the drainage. Finally, a routine drainage tube was placed after surgery. A 14 F drain was placed inside the wound for three days.

## 6 Follow up and outcomes

Postoperative examination showed significant decrease in inguinal lump size to 2 × 0.5 × 0.5 cm (Fig. 8). The backpain was immediately relieved (VAS 1). The patient was discharged after three days. In the first follow-up visit (7 days after surgery), there was no discharge from the wound, and the mass on the groin disappear. The back pain was alleviated, and the patient had returned to his normal activity without any complaint. MRI examination showed that the size of the abscess was markedly diminished compared ti the preoperative MRI (Fig. 9). The postoperative ODI score showed that the patient has minimal disability and normal function in the JOA score. The patient continues to consume the anti-tuberculosis drug for 6 months.

## 7 Discussion

As mentioned, the indication of surgical decompression of tuberculous spinal abscess is the failure of improvement or deterioration in neurological status during anti-tuberculous therapy, as most tuberculous abscess resolves with anti-tuberculous therapy. In our patient, surgical decompression was indicated due to the presence of positive leg raise tests in both legs, indicating presence of abscess compression in the iliopsoas muscle.

The Oswestry Disability Index is a common disability index to measures adult with spinal disorders. It’s considered as a gold standard for measuring disability and quality of life for adult with a low back pain. The ODI can be used to assess both chronic and acute conditions.

There are some approaches in performing surgery for sacral mass, including anterior, posterior, and presacral approaches. Anterior approach has the threat of vascular or neural damage. The presacral route, by using an avascular axial corridor to achieve vertebral access, has the advantage of sparing the posterior musculature, ligaments, and neural elements that are encountered during posterior approaches, as well as avoiding dissection and retraction of major vessels and the intra-abdominal viscera as with anterior approaches [12].

We confirmed the completeness of the drainage by the presence of clear fluid after irrigation of abscess, and its caseous material had been removed thoroughly. The success of this approach depends on the time of surgery. When surgery is delayed, the granulation tissue has formed, and in this condition, the anterior abdominal approach is required [13].

Research by Zhang et al. [10] suggested that MIS is suitable for most lumbosacral tuberculosis. However, it is not able to correct spinal deformity and relieve canal compression. Nevertheless, fortunately, the spinal canal has a relatively large volume, and the peripheral nerves in the canal have a relatively good tolerance for compression. Therefore, nerve compression rarely occurs in lumbosacral tuberculosis, making the MIS as an appropriate choice for most cases. The MIS has dual roles in both patients with nerve symptoms with and without kyphotic deformity. In patients without kyphosis deformity and severe nerve symptoms, MIS will make future open surgery safer. In patients with kyphosis and severe nerve symptoms, MIS can be performed to observe how the patient responds, in which when there is improvement during MIS, the non-open technique can be retained and that open surgery should be performed when the deterioration occurs. Although MIS cannot correct the kyphosis deformity, it can quickly improve patient’s general condition, remove the abscess and create right conditions for second stage of open corrective surgery when indicated [10].

Lumbosacral tuberculosis is adjacent to the rectum, iliac vessel, and ureter, where the abscess is often formed. Therefore, thorough removal of the diseased tissue is a highly demanding task during surgery. The tissue fragility, risk of vascular injury, and the incidence of retrograde ejaculation are increased by the inflammation in the surrounding tissue. This adding the consideration not to perform a thorough elimination of granulation tissue in front of and bilateral the vertebra to avoid the incidence of the vertebral injury. However, the abscess fluid must be evacuated as possible, and the necrotic tissue between the vertebrae and invading into the vertebral canal should be eliminated in order to enhance bone fusion and accelerate functional recovery [14].

The indications of MIS are unstable spine caused by vertebral destruction and deformity, nerve function injury, or paraspinal abscess [15]. The indication of MIS in our patient is the presence of paravertebral abscess formation extending to the right psoas muscle and inguinal region. When MIS is used, anatomical studies and radiological imaging is used instead of neurophysiological monitoring to avoid the early complication related to MIS [15]. This was what we performed to the patient in our case report, in which we used intraoperative fluoroscopic guidance when inserting the arthroscopic trochar into the abscess.

Some prerequisites of MIS must be fulfilled before the procedure is performed. These preoperative prerequisites are the fact that the preoperative anti-tuberculosis reaction was well, the preoperative imaging showed that the abscess formed can be evacuated although not entirely through a minimally invasive channel, and stable internal fixation for spinal reconstruction. This indicates that a preoperative regular anti-tuberculosis treatment is crucial before the surgical debridement is performed.

MIS using the presacral approach as described in AxiaLIF system is safe and effective. However, there are some limitations to the AxiaLIF procedure that must be considered. This approach requires surgeons to become familiar with presacral anatomy because the entire procedure is visualized under fluoroscopy with no direct observation of the vertebrae. The possible complication of this approach is bowel perforation, although rare. This complication can be avoided with appropriate preoperative patient preparation and meticulous surgical technique. Preoperative imaging should be thoroughly evaluated, with emphasis on perirectal fat pad thickness, identification of the rectum/sacrum interface, aberrant vasculature, and anticipated trajectory. Preoperative patient preparation includes mechanical bowel cleansing to enhance rectal pliability during blunt dissection and to lower contamination risk in the event of bowel injury. Administration of broad-spectrum intravenous antibiotics before the procedure will further lower the contamination risk. A precise initial incision followed by gentle blunt dissection with the finger allows for safe entry into the presacral area. Special attention to potential bowel complications should be given to women because the presacral width is narrower compared with males [12].

The Oswestry Disability Index is considered as a gold standard for measuring disability and quality of life for impairment adult with a low back pain. It consist of 10-point patient-reported questionnaire. The 10-point are pain intensity, ease of personal care, lifting, working, sitting, standing, sleeping, sex life, social life and travelling [16]. Japanese Orthopaedic Association (JOA) score is a score that used in patients with cervical compressive myelopathy to assess the severity of its clinical symptoms [17].

One of the goals of spinal tuberculosis treatment is to improve the patient’s quality of life. The advantages of MIS are a more favorable postoperative recovery without extensive soft tissue dissection and less estimated blood loss, and shorter hospital stay and operative time. Our patient stayed for three days in the hospital and discharged after the wound drain withdrew [15]. Case series by Arora et al. [18] showed that minimally invasive technique for abscess evacuation of lumbosacral abscess was a safe option, and compared to other approaches this transpedicular approach appears to be safer. The key to success of this approach is early diagnosis and early treatment to avoid the alteration of the liquid abscess into the granulomatous tissue [17,18]. Wang et al. [15] also noted in their study that MIS appeared to be a promising new treatment for lumbar tuberculosis. We concluded that MIS was a safe procedural and can be a choice for surgical evacuation of paraspinal abscess formed in lumbosacral tuberculosis. Preoperative prerequisites must be fulfilled before the procedure was performed in order to achieve the success of this method of surgery.

## 8 Patient perspective

After surgical debridement and abscess evacuation, antituberculous treatment was continued post-operatively. The patient had recovery of all back pain by two weeks postoperatively. Acid Fast Bacillus staining for tuberculous bacteria was decisive in the pus evacuated during surgery. At 3-months follow-up, patient was asymptomatic and can return to his normal activities.

## Declaration of Competing Interest

The authors declare no conflicts of interest.

## Funding

The authors report no external source of funding during the writing of this article.

## Ethical approval

Ethical approval was not required in the treatment of the patient in this report.

## Consent

Written consent has been received from the subject.

## Author contribution

Singkat Dohar Apul Lumban Tobing contributes to the study concept or design and data analysis or interpretation.

Dody Kurniawan contributes in the data collection, analysis and interpretation, and writing the paper.

## Registration of research studies

NA.

## Guarantor

Singkat Dohar Apul Lumban Tobing is the sole guarantor of this submitted article.

## Provenance and peer review

Not commissioned, externally peer-reviewed.
